# Characterizing dynamic behaviors of three-particle paramagnetic microswimmer near a solid surface

**DOI:** 10.1186/s40638-017-0076-0

**Published:** 2017-11-16

**Authors:** Qianqian Wang, Lidong Yang, Jiangfan Yu, Li Zhang

**Affiliations:** 1 Department of Mechanical and Automation Engineering, The Chinese University of Hong Kong, Shatin, Hong Kong SAR, China; 2 Chow Yuk Ho Technology Centre for Innovative Medicine, The Chinese University of Hong Kong, Shatin, Hong Kong SAR, China; 3 Shenzhen Research Institute, The Chinese University of Hong Kong, Shenzhen, 518172 China

**Keywords:** Swimming microrobot, Magnetic actuation, Boundary effect, Low Reynolds number, Dynamic behavior

## Abstract

Particle-based magnetically actuated microswimmers have the potential to act as microrobotic tools for biomedical applications. In this paper, we report the dynamic behaviors of a three-particle paramagnetic microswimmer. Actuated by a rotating magnetic field with different frequencies, the microswimmer exhibits simple rotation and propulsion. When the input frequency is below 8 Hz, it exhibits simple rotation on the substrate, whereas it shows propulsion with varied poses when subjected to a frequency between 8 and 15 Hz. Furthermore, a solid surface that enhances swimming velocity was observed as the microswimmer is actuated near a solid surface. Our simulation results testify that the surface-enhanced swimming near a solid surface is because of the induced pressure difference in the surrounding fluid of the microagent.

**Electronic supplementary material:**

The online version of this article (10.1186/s40638-017-0076-0) contains supplementary material, which is available to authorized users.

## Introduction

Microswimmers remotely actuated by magnetic fields have been considered as promising microrobotic tools because of their great potential in biomedical applications [1], such as targeted therapy [2], drug delivery [3, 4] and minimally invasive surgery [5]. Various designs of microswimmers combined with diverse magnetic actuation strategies have been proposed [6–10]. Among them, inspired by *E. coil* bacterial, helical microswimmer has drawn attention of many researchers. For propulsion of helical microswimmers, rotating magnetic fields are widely used for the generation of corkscrew motion at low Reynolds number. It was reported that, actuated by a rotating magnetic field, the “artificial bacterial flagella” (ABF) perform versatile swimming behaviors and can act as effective tools for cargo transport and micromanipulation tasks [11–15]. These ABF were fabricated using self-scrolling technique [11], 3-D direct laser writing [14], glancing angle deposition technique [16, 17], DNA-based flagellar bundles [18], and so on. The dynamics of such helical swimmers have been studied systematically below, near and higher than the step-out frequency. For instance, to perform corkscrew motion with continuous rotation, usually a magnetic helical swimmer should be actuated with an input frequency that is below its step-out frequency, whereas the actuation with a frequency that is higher than the step-out frequency will lead to a so-called “jerky motion” [19, 20], i.e., the combination of a rotation with stops and backward motions [21], which results in a decrease in its translational velocity [12, 22–24]. Interestingly, Ghosh et al. [24] reported that a helical microswimmer could exhibit bistable behaviors under an external field near the step-out frequency, showing random switch between two configurations, i.e., propulsion or tumbling motion.

Unlike the propulsion of tiny structures with chirality in low Reynolds number regime, it has been demonstrated recently that randomly shaped microswimmers can also be actuated effectively using a rotating magnetic field [25, 26]. These microswimmers are obtained using iron oxide nanoparticle aggregations with varied shapes based on hydrothermal carbonization. Alternatively, Cheang et al. [27] reported that achiral three-particle microswimmers exhibit controlled swimming motion under a rotating magnetic field. These microswimmers consist of three polystyrene microparticles embedded with paramagnetic or ferromagnetic nanoparticles, and varied swimming behaviors are triggered because of their different magnetic properties, despite the geometrical similarity.

It is notable that recent studies of three-particle microswimmers focus on swimming behaviors in fluid with negligible boundary effects [27–29]; however, their swimming behaviors near a solid surface can be significantly affected due to the boundary effect. Previously, the boundary effects were reported on both natural swimming organisms and artificial swimmers. The influence of solid boundaries has been observed and analyzed for *E. coil* bacteria [30, 31], and spermatozoa self-organized into dynamic vortices resembling quantized rotating waves on a planar surface [32]. A solid surface affects swimming direction of ABF, resulting in drifting behaviors [14], and wobbling motion of the ABF enhances the sidewise drift due to wall effects [33]. Simulation results indicate that microswimmer exhibits enhanced mobility when swimming between inclined rigid boundaries [34], and a surface can deform the induced streamlines of a rotating microagent [35].

**Fig. 1:**
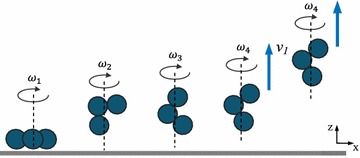
Schematic of the dynamic behaviors of a three-particle microswimmer under a rotating magnetic field. Black dashed line and arrows refer to the rotation axis and direction with angular velocity $\omega _1<\omega _2<\omega _3 <\omega _4$, blue arrows refer to velocity with $v _1>v_2$. The microswimmer exhibits simple rotation ($\omega _1$) and propulsion under different input frequencies ($\omega _2$, $\omega _3$, $\omega _4$). Gray rectangle refers to a solid surface

 Here, we report the dynamic behaviors of a paramagnetic three-particle microswimmer, which is actuated near a solid surface using a rotating magnetic field. With rotation axis of the magnetic field perpendicular to the horizontal surface, the microswimmer exhibits simple rotation when the input frequency is below 8 Hz, whereas it shows propulsion when subjected to a frequency between 8 and 15 Hz (Fig. 1). Furthermore, enhanced swimming velocity can be achieved if the microswimmer exhibits propulsion near the surface, because of the induced pressure difference in the surrounding fluid of the microswimmer. While with the rotation axis of the field parallel to the surface, the microswimmer exhibits low-frequency tumbling (1–3 Hz) and wobbling (3–15 Hz). The main contributions of this work include the following two aspects. First, a mathematical model is proposed for the analysis of dynamic poses under different input frequencies. Second, simulation results show that the induced pressure near a surface can enhance swimming velocity of a three-particle microswimmer, which are validated by experimental results.

The remaining parts of this paper are structured as follows. Mathematical modeling and simulations of the microswimmer are presented in Methods section. Then, in section Results and discussion, we discuss the dynamic behaviors of this microswimmer, and the experimental results are analyzed as well. Finally, *Conclusions* are given in the last section.

## Methods

### Mathematical modeling

**Fig. 2:**
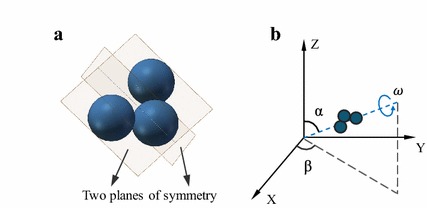
Structure of the microswimmer and the applied magnetic field. **a** Microswimmer is treated as a rigid structure with two mutually perpendicular planes of symmetry. **b** Schematic of the rotating magnetic field with constant flux density. Blue dashed line and arrow refer to the normal line and rotation direction of the magnetic field, respectively. Pitch angle $\alpha$ is between the normal line and *Z*-axis, and yaw angle $\beta$ is between *X*-axis and projection of the normal line in the *XY*-plane

The three-particle microswimmer is treated as a rigid structure with two perpendicular planes of symmetry, forms an achiral structure (Fig. 2a). It is placed on a solid surface and actuated by a rotating magnetic field (Fig. 2b).

#### Motion at low Reynolds numbers

The hydrodynamics of the microswimmer in low Reynolds number regime can be described by the Stokes equations:

1 $$\eta \nabla ^2{\mathbf {u}}-\nabla p=0$$

2 $$\begin{aligned} \nabla \cdot u=0 \end{aligned}$$

where *p* is pressure and *u* is moving velocity of the fluid. The relationship of external force ${\varvec{F}}$ together with torque $\varvec{\tau }$ and translational velocity $\varvec{V}$ together with angular velocity $\varvec{\omega }$ is described as [36]:

3 $$\begin{aligned} \left[ \begin{array}{lll} \varvec{V} \\ \varvec{\omega } \end{array} \right] = \left[ \begin{array}{ccc} \varvec{K} &{} \varvec{C_o}\\ \varvec{C_o^T} &{}\varvec{\Omega _o} \end{array} \right] \left[ \begin{array}{ccc} \varvec{F} \\ \varvec{\tau } \end{array} \right] \end{aligned}$$

where $\varvec{K}$ is the translation tensor and $\varvec{\Omega _o}$ is the rotation tensor. $\varvec{C_o}$ is the coupling tensor, representing coupling of translational and rotational motions of a microagent. For the microagent in Fig. 2a, the matrices $\varvec{K}$, $\varvec{\Omega _o}$ and $\varvec{C_o}$ are given by

4 $$\begin{aligned} {\mathbf {K}} = \left[ \begin{array}{lll} K_1 &{} 0 &{} 0 \\ 0 &{} K_2 &{} 0 \\ 0 &{} 0 &{} K_3 \\ \end{array} \right] ,\quad \mathbf {\Omega _o} = \left[ \begin{array}{ccc} \Omega _1 &{} 0 &{} 0 \\ 0 &{} \Omega _2 &{} 0 \\ 0 &{} 0 &{} \Omega _3 \\ \end{array} \right] ,\quad \mathbf {C_o} = \left[ \begin{array}{ccc} 0 &{} 0 &{} 0 \\ 0 &{} 0 &{} C_{23} \\ 0 &{} C_{32} &{} 0 \\ \end{array} \right] \end{aligned}$$

#### Magnetic actuation

The magnetic force and torque exerted on the microswimmer are given by:

5 $$\begin{aligned} \varvec{F}=(\varvec{m}\cdot \nabla )\varvec{B} \end{aligned}$$

6 $$\begin{aligned} \varvec{\tau }=\varvec{m} \times \varvec{B} \end{aligned}$$

where $\varvec{m}$ is the induced magnetic dipole moment and $\varvec{B}$ is the flux density of the magnetic field. Here we have $\varvec{F}=0$ because the applied magnetic field has uniform flux density. The angular and translational velocity of the microswimmer due to induced magnetic torque is denoted by

7 $$\begin{aligned} \varvec{\omega }=\varvec{\Omega _o}(\varvec{m}\times \varvec{B}) \end{aligned}$$

8 $$\begin{aligned} \varvec{V}=\varvec{C_o}(\varvec{m}\times \varvec{B}) \end{aligned}$$

Two equations above indicate that if the coupling tensor $\varvec{C_o}$ is nonzero, a rotating microswimmer can exhibit translational velocity.

**Fig. 3:**
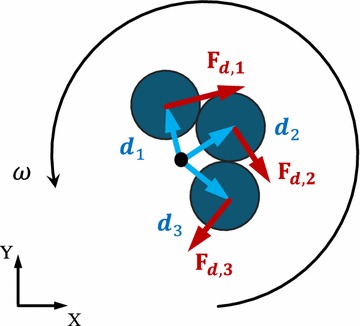
Schematic of the microswimmer actuated with simple rotation. Black dot and arrow refer to the rotation axis and direction of the microswimmer with angular velocity $\omega$, respectively. Three blue arrows $d_i$ are the vector positions of the three particles, and red arrows $F_{d,i}$ are the drag forces exerted on each particle (*i* = 1, 2, 3)

Next, we show the two torques (i.e., drag torque and magnetic torque) counterbalanced with each other. When the pitch angle $\alpha$ is 0, $\varvec{m}$ and $\varvec{B}$ are both in a plane perpendicular to the *Z*-axis (Fig. 3), making the angular velocity $\varvec{\omega } = [0\quad 0 \quad \omega _z]^T$ and magnetic torque $\varvec{\tau _m} = [0\quad 0 \quad \tau _{mz}]^T$. The induced magnetic torque can be treated as the torque exerted on a chain that consists of three spherical particles [37], as expressed by

9 $$\begin{aligned} \tau _{mz}=\frac{3}{4}\pi a^3 \mu _0 \chi ^2 B^2 \sin (2\theta ) \end{aligned}$$

where *a* is radius of the particle, $\mu _0$ is the vacuum permeability, $\chi$ is the particle susceptibility and $\theta$ is the phase lag between external field and induced dipole moment. If the microswimmer is actuated with steady rotation, the phase lag must satisfy the condition $\sin (2\theta )<1$ [37]. The drag torque $\varvec{\tau _r}$ due to hydrodynamic interaction can be obtained by combining torque from each particle individually [38]. Similarly, we have $\varvec{\tau _r} = [0\quad 0 \quad \tau _{rz}]^T$. For each particle, the drag torque is given by

10 $$\begin{aligned} \varvec{\tau _{rz,i}}=\varvec{d_i}\times \varvec{F_{d,i}} \end{aligned}$$

11 $$\begin{aligned} \varvec{F_{d,i}}=D_d\varvec{V_i}=D_d(\varvec{\omega _z}\times \varvec{d_i}) \end{aligned}$$

where $\varvec{d_i}$ and $\varvec{F_{d,i}}$ are vector position and drag force of the $i-th$ microparticle, and $D_d$ is the drag force coefficient, respectively. For spherical microparticles without any effects from boundary $D_d=-6\pi \eta a$. The total drag torque is given by

12 $$\begin{aligned} \varvec{\tau _{rz}}=\sum _{n=1}^{3}\varvec{d_i}\times \varvec{F_{d,i}} = D_d \varvec{\omega _z}\sum _{n=1}^{3} {d_i^2} \end{aligned}$$

Eq.12 shows that larger $\sum _{i=1}^{3}d^2_i$ leads to larger drag torque under the same input frequency.

#### Pose change frequency

The microswimmer undergoes constant magnetic torque due to uniform magnetic flux density. However, the drag torque is in dependence on the input frequency of magnetic field and rotation pose of the microswimmer. Next, from the torque-balance perspective, we show how swimming behaviors of our microswimmer varied by increasing the input frequency. The phase lag for a given input frequency and magnetic flux density is [37]

13 $$\begin{aligned} \sin (2\theta )=\frac{96\eta \omega }{\mu _0\chi ^2 B^2\ln (\frac{3}{2})} \end{aligned}$$

In order to balance the two torques, term $\sum _{i=1}^{3}d^2_i$ in Eq. 12 must change its value corresponding to different input frequencies, which results in different rotation poses of the microswimmer. However, the adjustable range of this term has limitation. Let us consider two cases of the microswimmer under actuation, i.e., simple rotation and propulsion. We simplify the microswimmer as an isosceles triangle with two sides of identical length *L* and the included angle $\gamma$. Since we only consider microswimmer with two perpendicular planes of symmetry, $\gamma$ in our analysis is set to be $\pi /3<\gamma <\pi$.

**Fig. 4:**
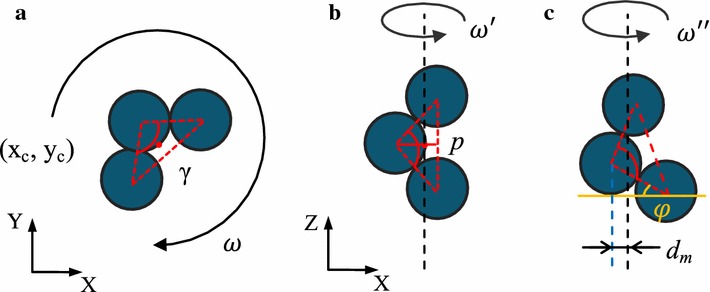
**a** Schematic of the microswimmer actuated with simple rotation. Rotation axis of the microswimmer coincides with centroid of the simplified isosceles triangle (red dash lines). The red dot refers to the projection of the rotation axis as well as the centroid with coordinate ($x_c$,$y_c$). **b** Rotation axis of the microswimmer coincides with the longest side of the simplified isosceles triangle. **c** Dynamic behaviors with pose angle $\varphi$ and distance $d_m$. Black arrows and dash lines refer to the angular velocity and rotation axis of the microswimmer, respectively. **b**, **c** Have the same coordinate

First, we assume that the microswimmer is actuated with simple rotation as shown in Fig. 4a. From the top view, the rotation axis is a dot with coordinate $(x_r,y_r)$. From geometrical perspective, we have

14 $$\begin{aligned} \sum _{n=1}^{3} {d_i^2}=(x_i-x_r)^2 + (y_i-y_r)^2 \end{aligned}$$

where $(x_i,y_i)$ is the coordinate of the $i-th$ particle’s center. The minimal value of $\sum _{i=1}^{3}d^2_i$ exists if the rotation axis passes through the centroid of the simplified isosceles triangle, given by

15 $$\begin{aligned} (x_c,y_c)=\left( \frac{\sum _{n=1}^{3}x_i}{3},\frac{\sum _{n=1}^{3}y_i}{3}\right) \end{aligned}$$

Substituting Eq. 15 into Eq. 14 yields

16 $$\sum _{n=1}^{3}d^2_{i{\rm min}} = \frac{2}{3}L^2 (2+\sin \gamma -\cos \gamma)$$

17 $$\begin{aligned}&\quad \sum _{n=1}^{3}d^2_{i{\rm min}}\in (1.58 L^2, 2.28 L^2) \end{aligned}$$

Then, we assume the microswimmer is actuated with propulsion as shown in Fig. 4b. In this scenario, the minimal value exists if the rotation axis is parallel to the longest side of the triangle, which is the side respected to angle $\gamma$ since $\gamma >\pi /3$. Calculation results show that the minimal value exists when the rotation axis passes through the point *p*, a point of trisection of the height with respect to the longest side. Similarly, we have

18 $$\sum _{n=1}^{3}d^2_{i{\rm min}} = \frac{2}{3} L^2 \cos ^2\frac{\gamma }{2}$$

19 $$\begin{aligned}&\quad \sum _{n=1}^{3}d^2_{i{\rm min}} \in \left( 0,\frac{1}{2}L^2\right) \end{aligned}$$

The analysis results above, in particular Eqs. 17 and 19, show that with the same input frequency, drag torque becomes smaller if the microswimmer exhibits propulsion rather than simple rotation. Finally, let us consider a specific case. We increase the input frequency continuously, at first, the microswimmer exhibits simple rotation, and then, it tends to change its actuation behaviors toward reducing the drag torque. The only feasible method is to reduce the distance (term $\sum _{i=1}^{3}d^2_i$ in Eq. 12) between each microparticle and the rotation axis. Therefore, the microswimmer has to change from simple rotation to propulsion when the input frequency is higher than a certain value $\omega _{c}$, and here we name $\omega _{c}$ as the pose-change frequency. When the angular velocity is high enough, the propulsion force of the microswimmer is larger than the combination of gravitational force and buoyancy, so that it will swim. A switch from simple rotation to propulsion can be realized by increasing the input frequencies with a value higher than $\omega _{c}$. For example in Fig. 1, $\omega _1$ is below $\omega _{c}$ while $\omega _2$, $\omega _3$, $\omega _4$ are higher than $\omega _{c}$.

Besides the two specific scenarios shown in Fig. 4a, b, other dynamic behaviors can be realized as well. As shown in Fig. 4c, we define the simplified triangular has an angle $\varphi$ with *X*-axis and the distance between the vertex and rotation axis is $d_m$. These two parameters are able to represent the propulsion pose with rotation axis. Here the $\sum _{i=1}^{3}d^2_i$ is calculated as

20 $$\begin{aligned} \sum _{i=1}^{3}d^2_i = 3d_m^2 + L^2[\cos ^2(\varphi )+\cos ^2(\varphi -\gamma )]-2Ld_m[\cos (\varphi )+\cos (\varphi -\gamma )] \end{aligned}$$

For a given microswimmer $\gamma =\pi /2$, if we define $d_m=\sigma L$ ($0 \le \sigma \le 1$) and Eq. 20 can be simplified as

21 $$\begin{aligned} \sum _{i=1}^{3}d^2_i = L^2[3\sigma ^2 + 1 -2\sigma (\sin \varphi + \cos \varphi )] \end{aligned}$$

**Fig. 5:**
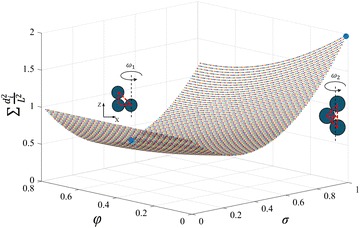
Distribution of the value of $\sum _{i=1}^{3}d^2_i$. The minimum and maximum values are marked with the corresponding rotation poses. The two insets have the same coordinate

The range of $\varphi$ is set to be $0\le \varphi \le \pi /4$, while $\pi /4 \le \varphi \le \pi /2$ results in the same value of $\sum _{i=1}^{3}d^2_i$ because of the symmetry of the model. We use *MATLAB* to calculate the distribution of the value in Eq. 21, and the results are shown in Fig. 5. The maximum value exists when $\sigma = 1$ and $\varphi =0$, corresponding to the pose with angular velocity $\omega _2$ in Fig. 1. Interestingly, the minimum value is $\sum _{i=1}^{3}d^2_i=L^2 /3$ when $\sigma = 0.47$ and $\varphi = \pi /4$, which also proves that minimal value exists when the rotation axis is parallel to the longest side of the simplified triangle model.

### Simulations

**Fig. 6:**
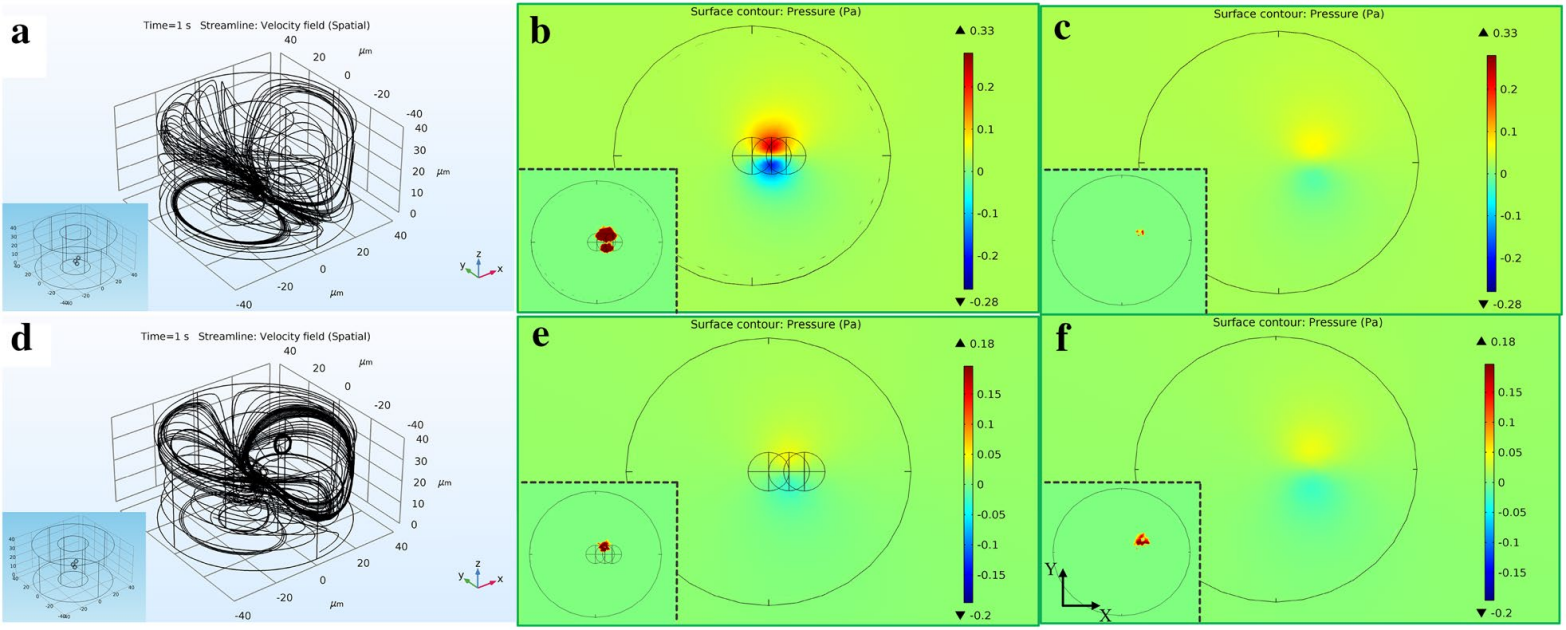
Simulation of the microswimmer rotates above a no-slip wall. Rotation axis is defined as *Z-*axis; numbers represent dimensions in micrometer. The microswimmer with two different boundary conditions is modeled **a** $0.75 \,\upmu \hbox {m}$ and **d** $20.75 \,\upmu \hbox {m}$ above the no-slip wall, as shown in the insets of **a** and **d**, respectively. **a**, **b** The streamlines generated by the microswimmer. **b**, **c**, **e**, **f** Pressure induced by rotation of the microswimmer at a frequency of 10 Hz in a plane $0.5 \,\upmu \hbox {m}$ below **b**, **e** and above **c**, **f** the microswimmer, **b**, **c** are with the microswimmer $0.75 \,\upmu \hbox {m}$ above the no-slip wall, and **e**, **f** are $20.75 \,\upmu \hbox {m}$ above the no-slip wall. The color legends in the main frame illustrate the magnitude of pressure (Pa). The red areas of insets in** b**,** e** are with pressure higher than 0.1 Pa, and those in **e**, **f** are higher than 0.04 Pa. **b**, **c**, **e**, **f** with all insets have the same coordinate

**Fig. 7:**
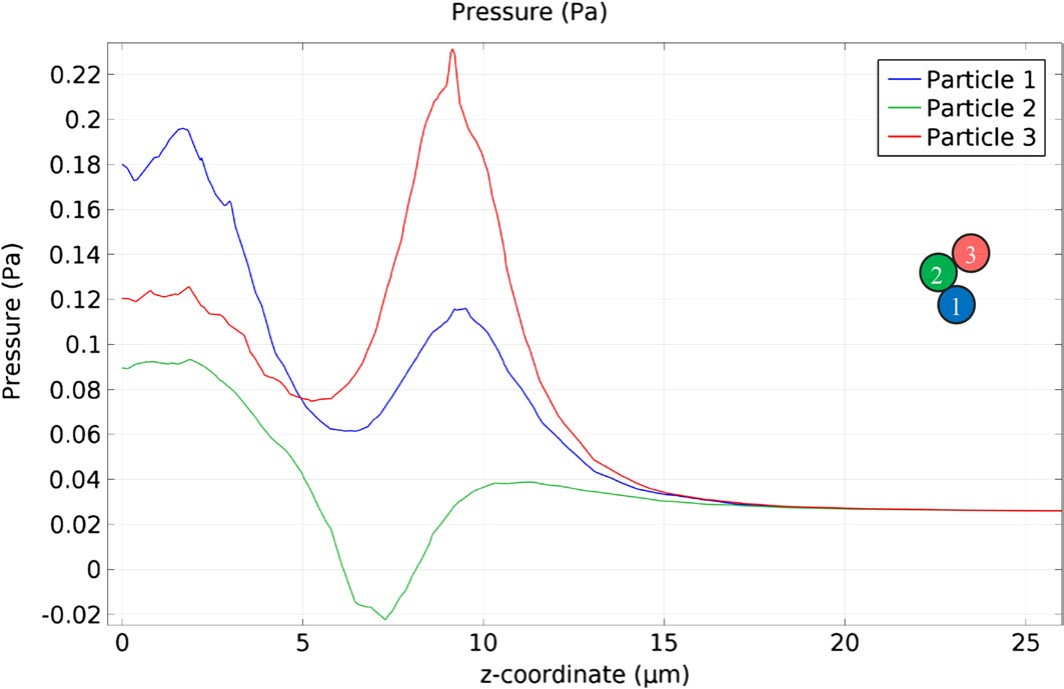
Simulation results of the pressure distribution. Pressure distribution near the microswimmer. Three lines denotes the pressure near the three particles in blue, green and red, respectively

To simulate and understand how a solid surface affects swimming behaviors, two finite element method (FEM) models are established using *COMSOL Multiphysics* (two insets in Fig. 6a, d) to investigate the induced fluid flows (Fig. 6a, b) and pressure (Fig. 6b, c, e, f) by the rotating microswimmer. The microswimmer is modeled as three spheres with a diameter of 4.5 $\upmu \hbox {m}$ and an angle $\gamma = \pi /2$, and set to be actuated in water at a frequency of 10 Hz. A solid surface is modeled as a no-slip wall at the bottom. Simulations consist of two cases: Fig. 6a–c are simulation results with microswimmer near (0.75 $\upmu \hbox {m}$) the surface, and Fig. 6d–f are results with it farther away (20.75 $\upmu \hbox {m}$) from the surface. After over ten full rotations, the induced pressure distribution and streamlines of the surrounding fluid are calculated. Figure 6a, b show that the microswimmer induces a net flow of fluid along the direction of the rotation axis, similar to the propulsion of a helical flagellum [15, 39]. The fluid impacts on the substrate, resulting in enhanced pressure [40]. For the case of rotation near the surface, the induced pressure difference between the area near the top and bottom space of the microswimmer is observed in Fig. 6b, c. However, such difference becomes negligible when the microswimmer 20.75 $\upmu \hbox {m}$ above the surface (Fig. 6e, f). The largest pressure difference around each particle is in the order of $10^{-2}$ Pa (Fig. 7). The affected area on the microswimmer is in the order of $10^1$ $\upmu \hbox {m}^2$ and the net force along *Z*-axis works out in piconewton range due to the pressure difference.

### The microswimmer and experimental setup

In our experiments, the microswimmer was obtained by direct sediment of paramagnetic microparticles colloidal suspensions (Spherotech PMS-40-10) in DI water. These microparticles have a density of 1.27 $\hbox {g}/\hbox {cm}^3$ and a diameter of 4–5 $\upmu \hbox {m}$ with a smooth surface. Sediment introduces randomness to the process, resulting in different structures. Nonetheless, the three-particle structures can be easily obtained and directly used in our experiments. During the magnetic actuation, we did not observe deformation of the swimmer by turning on and off the field, which indicates the link between two microparticles is fixed and stable.

**Fig. 8:**
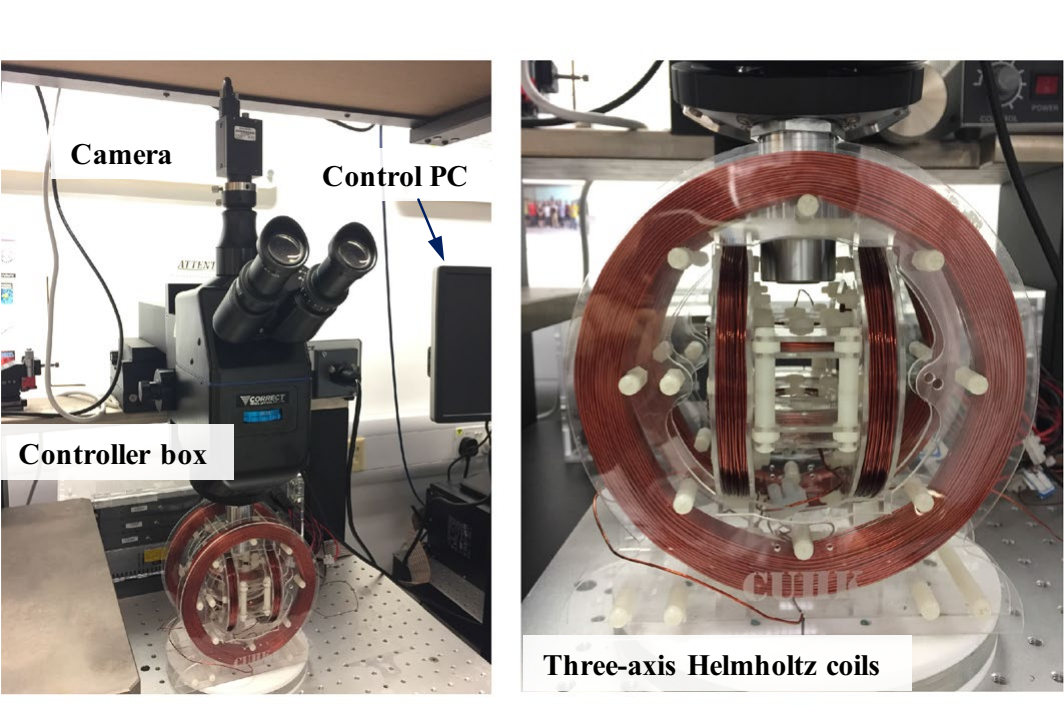
Magnetic actuation setup. Three-axis Helmholtz electromagnetic coils are applied for generating rotating magnetic field. A camera is mounted on the top of a light microscope for video recording. The actuation setup is controlled by using a PC and a controller box with three amplifiers and one power supply inside

Our electromagnetic coils setup consists of three orthogonally placed Helmholtz coil pairs, a swimming tank containing a Si substrate inside and a light microscope with a recording camera on the top. Rotating magnetic field is generated by the coil system (Fig. 8) actuated by three servo amplifiers (ADS 50/5 4-Q-DC, Maxon Inc.). The amplifiers are controlled by a *LabVIEW* program through an Analog and Digital I/O card (Model 826, Sensoray Inc.), frequency, field strength as well as yaw ($\beta$) and pitch angles ($\alpha$) can be adjusted through this program. Schematic of the magnetic field is shown in Fig. 2b. A swimming tank ($21 \hbox {mm} \times 21 \hbox {mm} \times 3 \hbox {mm}$) filled with DI water is placed in the middle of the coils, and the Si substrate inside provides a solid surface. The top camera records the motion of the microswimmer at a rate of 50 fps.

## Results and discussion

### The microrobot swims away from the solid surface ($\alpha =0^{\circ }$)

**Fig. 9:**
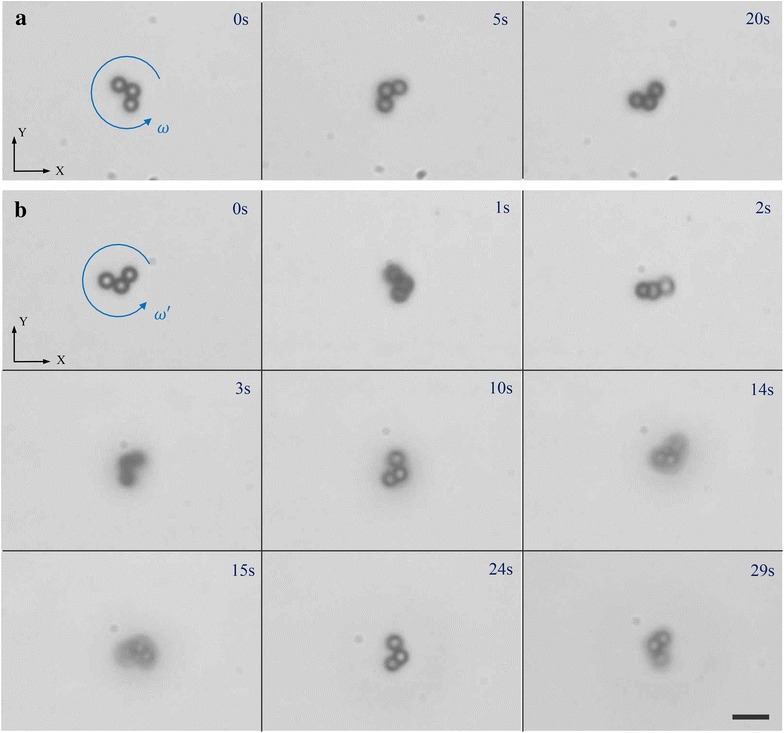
**a** Time-lapse images of the three-particle magnetic microswimmer which is actuated with simple rotation under a rotating magnetic field at a frequency of 7 Hz. **b** The microswimmer is actuated with dynamic behaviors (0–2 s) and steady propulsion (2–29 s) under a rotating magnetic field at a frequency of 9 Hz. At 0–2 s, the focal plane is on the substrate, and then, the focal plane is adjusted to a plane 20 $\upmu \hbox {m}$ (3–14 s) and 40 $\upmu \hbox {m}$ (15–29 s) above the substrate, respectively. Blue arrows refer to the rotation direction of the microswimmer. Scale bar is 10 $\upmu \hbox {m}$; all the images in **a**, **b** have the same scale bar

The microswimmer has been actuated at a frequency range from 1 to 16 Hz on a Si substrate in the tank. The flux density of the magnetic field maintains 9 mT during the experiments. When the input frequency is below 8 Hz, the microswimmer exhibits simple rotation and no translational velocity is observed (Fig. 9a), whereas it exhibits propulsion with varied poses when the input frequency is higher than 8 Hz (Fig. 9b). Experiment results show that 8 Hz is the pose-change frequency $\omega _{c}$. When the input frequency is below $\omega _{c}$ (8 Hz), the microswimmer exhibits simple rotation and the drag torque is small enough to be balanced by the magnetic torque. The projection of rotation axis in *XY*-plane is closing to the centroid of the simplified triangle gradually with increasing the input frequency, in order to reduce the drag torque (Fig. 4a). Such adjustment of rotation axis cannot affect the actuation pose (simple rotation) of the microswimmer. Equations 14–17 have shown the limitation of this adjustment method, which also explains why the microswimmer cannot maintain simple rotation with input frequency higher than $\omega _{c}$. While when the input frequency is higher than $\omega _{c}$ (8 Hz), the drag torque is affected by both the pose angle $\varphi$ and the distance $d_m$ (Fig. 4c). Different input frequencies of the magnetic field change the drag torque, and dynamic behaviors of the microswimmer are governed by the interplay of magnetic and resistant torques. The dynamic behaviors appear when turning on the magnetic field or changing the input frequency (see Additional file 1). As shown in the experimental results (Fig. 9b), after turning on the magnetic field the dynamic behaviors of the microswimmer last less than 2 s (0–2 s). After that the microswimmer exhibits steady rotation and propulsion (2–29 s).

**Fig. 10:**
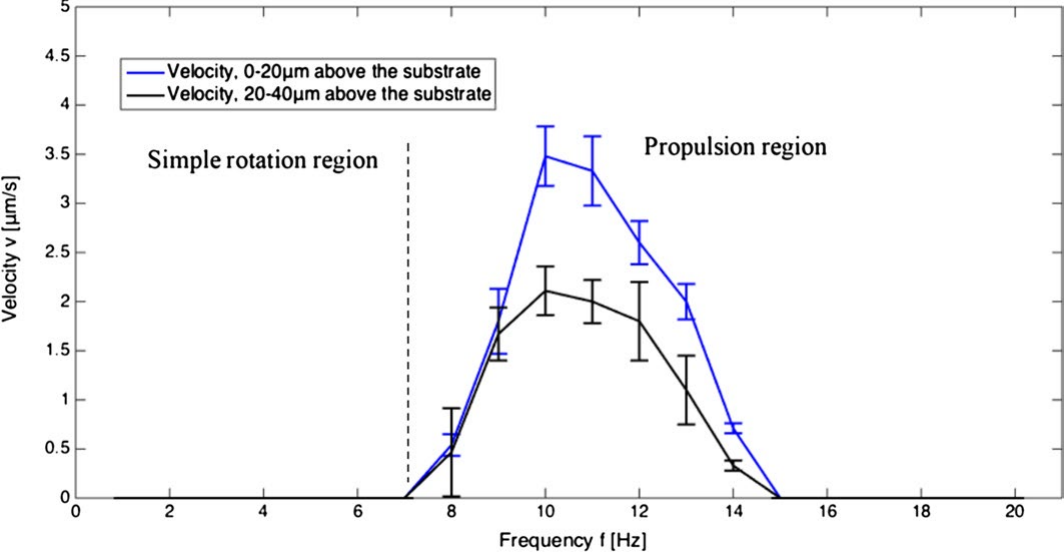
Velocity of the microswimmer against frequency. Velocity of the microswimmer against frequency of the applied magnetic field. Rotation axis is perpendicular to the solid surface ($\alpha =0^{\circ }$). Blue and black lines are the velocity in bottom space (0–20 $\upmu \hbox {m}$ above the substrate) and upper space (20–40 $\upmu \hbox {m}$ above the substrate), respectively. Error bars denote the standard errors from observation time that is used to calculate velocity

Swimming velocity of the microswimmer along the *Z*-axis is measured by a fixed distance $\Delta z$ divided by time $\Delta t$. It follows three steps. First, the focal plane of the microscope is set on the substrate, followed by recording and turning the magnetic field on. This step aims to record the starting time. Then, the focal plane is adjusted to 20 $\upmu \hbox {m}$ above the substrate. The microswimmer swimming across the focal plane is observed as it became in focus gradually and then out of focus. Finally, we find the best focusing frame from the recorded video to count the time $\Delta t$. Using this method, swimming velocity of the microswimmer in the space 0 to $20 \,\upmu \hbox {m}$ above the substrate (bottom space) is measured. The velocity in the space 20–40  $\upmu \hbox {m}$ (upper space) above the substrate is measured using the same method. After turning off the magnetic field, the microswimmer will gradually sink onto the substrate due to gravitational force. The swimming velocity against frequency in the bottom and upper space is depicted in Fig. 10.

**Fig. 11:**
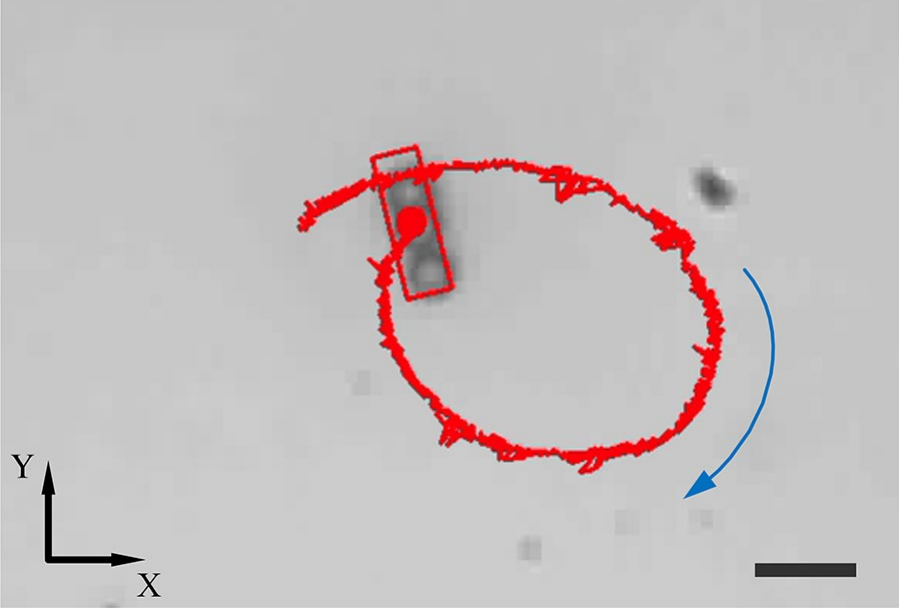
Steering of the microswimmer. Swimming trajectory (red line) of the microswimmer in a plane 25 $\upmu \hbox {m}$ above the substrate (top view). Blue arrow refers to the swimming direction, and red rectangular is applied for tracking the position of the microswimmer. Scale bar is 10 $\upmu \hbox {m}$

Next, we show magnetic steering of the microswimmer. It swims along the direction of +*Z*-axis after exerting field at a frequency of 10 Hz with $\alpha =0^{\circ }$. After lifting $25 \,\upmu \hbox {m}$ from the substrate, it can stay in focus by adjusting pitch angle $\gamma$ to $80^{\circ }$, showing negligible displacement along *Z*-direction (see Additiona file 1). The propulsive force has the same direction with the normal line of the applied magnetic field. In this scenario, gravitational force and buoyancy are balanced by the component of propulsive force on *Z-*axis. Steering can be done by adjusting the yaw angle $\beta$ of the field from 0$^{\circ }$ to 360$^{\circ }$ as shown in Fig. 11. The microswimmer did not show visible sidewise drift because of the absence of the boundary effect [33]. The propulsive force is measured based on the equilibrium of forces, which contains gravitational force, buoyancy and propulsive force. The gravitational force and buoyancy, respectively, are 1.82 and 1.43 pN, and the propulsive force is calculated to be 1.14 pN. Based on the simulation results, the net force generated by pressure difference is in the order of piconewton as well, which implies this net force can enhance swimming velocity. Figure 10 shows that the microswimmer has a higher swimming velocity in the bottom space (0–20 $\upmu \hbox {m}$ above the surface), which validates our calculation and simulation results.

### Actuation of the microrobot near the solid surface ($\alpha =90^{\circ }$)

**Fig. 12:**
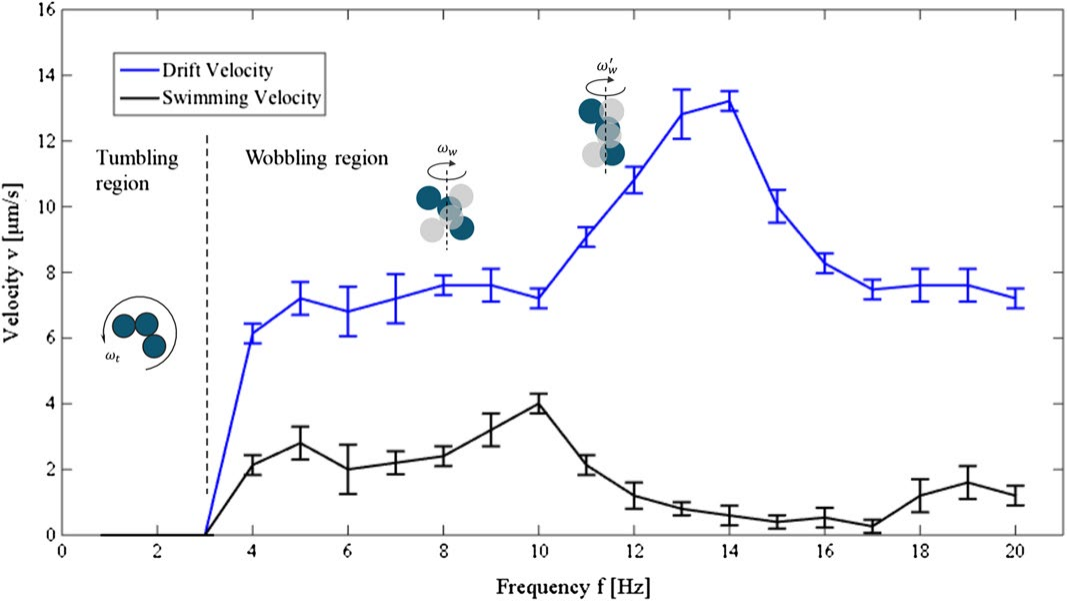
Velocity of the microswimmer near a solid surface. Swimming and drift velocity of the microswimmer with rotation axis parallel to the solid surface ($\alpha =90^{\circ }$). The microswimmer exhibits dynamic behaviors with increasing frequency. The error is from the pixel size of the camera and software *Image J*

The microswimmer has been actuated with pitch angle $\alpha =90^{\circ }$ (i.e. its rotation axis is parallel to the horizontal substrate) above the Si substrate. It shows frequency-dependent motion regimes, that is, tumbling (1–3 Hz) and wobbling (3–20 Hz). The plot of velocity verses frequency is depicted in Fig. 12. When input frequency is below 3 Hz, the microswimmer exhibits tumbling motion with 90$^{\circ }$ precession angle. After increasing the input frequency to be higher than 3 Hz, the microswimmer exhibits wobbling motion and the precession angle decreases continually under higher frequency. Previous studies show that dynamic regimes for the tumbling-to-wobbling transition of a magnetic microswimmer depend not only on the frequency of the magnetic field, but also on the geometry and easy axis orientation of the microswimmer [41]. In our experiments, when the microswimmer is actuated at a frequency range of 1–3 Hz, both the easy axis and the induced magnetic moment are oriented along the field direction. The easy axis and the induced magnetic moment rotate with a phase lag behind the magnetic field, resulting in tumbling motion of the microswimmer. After increasing the input frequency to be higher than 3 Hz, the drag torque increases and its interplay with the magnetic torque results in wobbling regime. During the experiments, the precession angle of the microswimmer decreases with increasing input frequency of the magnetic field, same as the theoretical prediction [41]. The swimming velocity reaches maximum under magnetic field at a frequency of 10 Hz, similar to the results shown in Fig. 10.

Drifting of the microswimmer occurs due to the boundary effects. The drag coefficient is constant for a certain sphere particle in bulk fluid, but the presence of a solid surface increases the drag on a body, which decreases with a growing distance between the microswimmer and the surface [42]. To be specific, a segment of the microswimmer closer to the surface exhibits larger drag than that farther away the surface, which causes the microswimmer drift sidewise, perpendicular to the rotation axis. Figure 12 also indicates that unlike the ABF in [33], the drift velocity of the microswimmer is not increasing linearly with the input frequency.

## Conclusions

In this paper, we demonstrate dynamic behaviors of a three-particle paramagnetic microswimmer near a solid surface. These dynamic behaviors are dependent on the input frequency of the rotating magnetic field, and varied actuation poses can be switched by adjusting the frequency. Simulations of the microswimmer near ($0.75\,\upmu \hbox {m}$) and far farther away ($20.75 \,\upmu \hbox {m}$) from a solid surface are investigated, which are in good agreement with the experimental results. Finally, the effects of a solid surface on swimming behaviors are proposed, i.e., enhancing swimming velocity when the microswimmer exhibits propulsion perpendicular to the horizontal surface and causing sidewise drift when it is actuated parallel to the surface. Future studies will focus on the motion control of the microswimmer in biofluids with different viscosities.

## Additional file

**Additional file 1.** This video demonstrates the microswimmer actuated under a rotating magnetic field at a frequency of 7 Hz and 9 Hz, and magnetic steering of the microswimmer.

## References

[1] Sitti M, Ceylan H, Hu W, Giltinan J, Turan M, Yim S, Diller E (2015). Biomedical applications of untethered mobile milli/microrobots. Proc IEEE.

[2] Nelson BJ, Kaliakatsos IK, Abbott JJ (2010). Microrobots for minimally invasive medicine. Annu Rev Biomed Eng.

[3] Vikram Singh A, Sitti M (2016). Targeted drug delivery and imaging using mobile milli/microrobots: A promising future towards theranostic pharmaceutical design. Curr Pharm Des.

[4] Yan X, Zhou Q, Yu J, Xu T, Deng Y, Tang T, Feng Q, Bian L, Zhang Y, Ferreira A (2015). Magnetite nanostructured porous hollow helical microswimmers for targeted delivery. Adv Funct Mater.

[5] Kummer MP, Abbott JJ, Kratochvil BE, Borer R, Sengul A, Nelson BJ (2010). Octomag: an electromagnetic system for 5-dof wireless micromanipulation. IEEE Trans Robot.

[6] Abbott JJ, Peyer KE, Lagomarsino MC, Zhang L, Dong L, Kaliakatsos IK, Nelson BJ (2009). How should microrobots swim?. Int J Robot Res.

[7] Peyer KE, Tottori S, Qiu F, Zhang L, Nelson BJ (2013). Magnetic helical micromachines. Chem-A Eur J.

[8] Peyer KE, Zhang L, Nelson BJ (2013). Bio-inspired magnetic swimming microrobots for biomedical applications. Nanoscale.

[9] Yu J, Xu T, Lu Z, Vong CI, Zhang L. On-demand disassembly of paramagnetic nanoparticle chains for microrobotic cargo delivery. IEEE Trans Robot. 2017;33(5). 10.1109/TRO.2017.2693999.

[10] Yang L, Wang Q, Vong C-I, Zhang L (2017). A miniature flexible-link magnetic swimming robot with two vibration modes: design, modeling and characterization. IEEE Robot Autom Lett.

[11] Zhang L, Abbott JJ, Dong L, Kratochvil BE, Bell D, Nelson BJ (2009). Artificial bacterial flagella: fabrication and magnetic control. Appl Phys Lett.

[12] Zhang L, Abbott JJ, Dong L, Peyer KE, Kratochvil BE, Zhang H, Bergeles C, Nelson BJ (2009). Characterizing the swimming properties of artificial bacterial flagella. Nano Lett.

[13] Tottori S, Zhang L, Qiu F, Krawczyk KK, Franco-Obregón A, Nelson BJ (2012). Magnetic helical micromachines: fabrication, controlled swimming, and cargo transport. Adv Mater.

[14] Tottori S, Zhang L, Peyer KE, Nelson BJ (2013). Assembly, disassembly, and anomalous propulsion of microscopic helices. Nano Lett.

[15] Zhang L, Peyer KE, Nelson BJ (2010). Artificial bacterial flagella for micromanipulation. Lab Chip.

[16] Ghosh A, Fischer P (2009). Controlled propulsion of artificial magnetic nanostructured propellers. Nano Lett.

[17] Fischer P, Ghosh A (2011). Magnetically actuated propulsion at low Reynolds numbers: towards nanoscale control. Nanoscale.

[18] Maier AM, Weig C, Oswald P, Frey E, Fischer P, Liedl T (2016). Magnetic propulsion of microswimmers with DNA-based flagellar bundles. Nano Lett.

[19] Helgesen G, Pieranski P, Skjeltorp AT (1990). Nonlinear phenomena in systems of magnetic holes. Phys Rev Lett.

[20] Helgesen G, Pieranski P, Skjeltorp A (1990). Dynamic behavior of simple magnetic hole systems. Phys Rev A.

[21] Sandre O, Browaeys J, Perzynski R, Bacri J-C, Cabuil V, Rosensweig R (1999). Assembly of microscopic highly magnetic droplets: magnetic alignment versus viscous drag. Phys Rev E.

[22] Gao W, Feng X, Pei A, Kane CR, Tam R, Hennessy C, Wang J (2013). Bioinspired helical microswimmers based on vascular plants. Nano Lett.

[23] Mahoney AW, Nelson ND, Peyer KE, Nelson BJ, Abbott JJ (2014). Behavior of rotating magnetic microrobots above the step-out frequency with application to control of multi-microrobot systems. Appl Phys Lett.

[24] Ghosh A, Paria D, Singh HJ, Venugopalan PL, Ghosh A (2012). Dynamical configurations and bistability of helical nanostructures under external torque. Phys Rev E.

[25] Vach PJ, Brun N, Bennet M, Bertinetti L, Widdrat M, Baumgartner J, Klumpp S, Fratzl P, Faivre D (2013). Selecting for function: solution synthesis of magnetic nanopropellers. Nano Lett.

[26] Vach PJ, Fratzl P, Klumpp S, Faivre D (2015). Fast magnetic micropropellers with random shapes. Nano Lett.

[27] Kei Cheang U, Lee K, Julius AA, Kim MJ (2014). Multiple-robot drug delivery strategy through coordinated teams of microswimmers. Appl Phys Lett.

[28] Cheang UK, Meshkati F, Kim D, Kim MJ, Fu HC (2014). Minimal geometric requirements for micropropulsion via magnetic rotation. Phys Rev E.

[29] Morozov KI, Mirzae Y, Kenneth O, Leshansky AM (2017). Dynamics of arbitrary shaped propellers driven by a rotating magnetic field. Phys Rev Fluids.

[30] Lauga E, DiLuzio WR, Whitesides GM, Stone HA (2006). Swimming in circles: motion of bacteria near solid boundaries. Biophys J.

[31] Lauga E, Powers TR (2009). The hydrodynamics of swimming microorganisms. Rep Prog Phys.

[32] Riedel IH, Kruse K, Howard J (2005). A self-organized vortex array of hydrodynamically entrained sperm cells. Science.

[33] Peyer KE, Zhang L, Kratochvil BE, Nelson BJ. Non-ideal swimming of artificial bacterial flagella near a surface. In: Robotics and Automation (ICRA), 2010 IEEE International Conference on. 2010, pp. 96–101.

[34] Ledesma-Aguilar R, Yeomans JM (2013). Enhanced motility of a microswimmer in rigid and elastic confinement. Phys Rev Lett.

[35] Zhou Q, Petit T, Choi H, Nelson BJ, Zhang L. Dumbbell fluidic tweezers for dynamical trapping and selective transport of microobjects. Adv Funct Mater. 2017;27(1). 10.1002/adfm.201604571.

[36] Happel J, Brenner H (1983). Low Reynolds number hydrodynamics: with special applications to particulate media.

[37] Biswal SL, Gast AP (2004). Rotational dynamics of semiflexible paramagnetic particle chains. Phys Rev E.

[38] Doi M, Edwards SF (1988). The theory of polymer dynamics.

[39] Liu B, Breuer KS, Powers TR (2014). Propulsion by a helical flagellum in a capillary tube. Phys Fluids.

[40] Wu G (2007). Fluid impact on a solid boundary. J Fluids Struct.

[41] Morozov KI, Leshansky AM (2014). Dynamics and polarization of superparamagnetic chiral nanomotors in a rotating magnetic field. Nanoscale.

[42] Brennen C, Winet H (1977). Fluid mechanics of propulsion by cilia and flagella. Annu Rev Fluid Mech.

